# Validation and Cultural Adaptation of the Serbian Version of the Cleveland Clinic Colorectal Cancer Quality of Life Questionnaire: A Comprehensive Psychometric Evaluation

**DOI:** 10.3390/healthcare13080937

**Published:** 2025-04-18

**Authors:** Vladimir Nikolic, Ljiljana Markovic-Denic, Lidija Masic, Aleksandar Sekulic, Stefan Kmezic, Djordje Knezevic, Aleksandar Radovanovic, Djordje Nektarijevic, Andrija Antic

**Affiliations:** 1 Institute of Epidemiology, Faculty of Medicine, University of Belgrade, 11129 Belgrade, Serbia; lj.denic@gmail.com; 2 Clinic of Digestive Surgery, University Clinical Center of Serbia, 11000 Belgrade, Serbia; lidijaamed@gmail.com (L.M.); sekulic038@yahoo.com (A.S.); kstefan1986@gmail.com (S.K.); dr.djordje.knezevic@gmail.com (D.K.); aleksaradovanovic95@gmail.com (A.R.); dr.nektar91@gmail.com (D.N.); drandrija.antic@gmail.com (A.A.); 3 Faculty of Medicine, University of Belgrade, 11129 Belgrade, Serbia

**Keywords:** colorectal cancer, quality of life, validation, CCF-CaQL

## Abstract

**Backround/Objectives:** Colorectal cancer presents a significant quality of life (QoL) challenge as a result of both the disease and its treatments. This study aimed to validate and culturally adapt the Cleveland Clinic Colorectal Cancer Quality of Life Questionnaire (CCF-CaQL) for Serbian-speaking colorectal cancer patients. **Methods:** The CCF-CaQL offers a detailed assessment of the physical, emotional, social, and functional impacts of the disease. This study, conducted at the University Clinical Center of Serbia, involved 150 colorectal cancer patients undergoing treatment. The translation and adaptation process followed the EORTC Quality of Life Group’s guidelines, ensuring cultural relevance and comprehensibility. Statistical analyses, including Cronbach’s alpha for internal consistency and Pearson’s correlation for concurrent validity, reliability, and known-groups validity, were performed using SPSS and R software. **Results:** The Serbian version of the CCF-CaQL maintains strong psychometric properties with high internal consistency (Cronbach’s alpha = 0.85) and significant correlations with the FACT-C questionnaire, confirming its validity. Known-groups validity showed distinct variations in QoL scores based on tumor location, stoma presence, and neoadjuvant therapy status, highlighting its sensitivity to different clinical conditions. **Conclusions:** The CCF-CaQL questionnaire has been skillfully translated, culturally adapted, and carefully validated through psychometric evaluations for Serbian patients diagnosed with colorectal cancer.

## 1. Introduction

Colorectal cancer (CRC) is one of the most common malignancies worldwide, representing a significant public health challenge, including in Serbia [1]. The global incidence of colorectal cancer is expected to continue rising as more countries undergo economic and demographic changes and adopt Western lifestyles [2]. The highest incidences have been reported in Europe, Australia, New Zealand, and North America [3,4]. Similarly, Serbia has seen a significant increase in colorectal cancer cases over the past three decades [5,6].

Evaluating Quality of Life (QoL) is crucial in cancer management, providing a more comprehensive view of the patient’s experience with the disease and its treatments compared to standard clinical outcomes [7]. This is particularly important in patients with colorectal cancer. CRC not only challenges patients with its direct health implications but also imposes a significant burden on their quality of life. This burden can be multidimensional, affecting physical, emotional, social, and economic aspects of life [8]. Existing evidence suggests that patients with colorectal cancer have a poorer quality of life compared to the general population, particularly in terms of physical health, emotional stability, and social interaction [9].

Among the specific instruments available for assessing quality of life in colorectal cancer patients, several instruments stand out: the European Organisation for Research and Treatment of Cancer’s (EORTC) Quality of Life Questionnaire for Colorectal Cancer (QLQ-CR29), which is designed to complement the core EORTC QLQ-C30 questionnaire by focusing on colorectal cancer-specific issues [10]; the North American Functional Assessment of Cancer Therapy (FACIT) questionnaire, FACT-C, which also addresses specific concerns related to colorectal cancer [11]; and the Cleveland Clinic Colorectal Cancer Quality of Life Questionnaire (CCF-CaQL), developed to provide a swift and comprehensive evaluation of the physical, emotional, social, and functional aspects of health in these patients. This latter tool aims to deliver a more precise assessment of quality of life, tailored specifically for individuals with colorectal carcinoma [12]. The original validation study was conducted across multiple international sites, including the United States, France, Germany, and Australia, and involved patients undergoing sphincter-saving surgery for low rectal cancer. Currently, the CCF-CaQL is validated for use in only two versions: English and Turkish [13].

At the time of study initiation, among the colorectal cancer-specific quality of life instruments, only the FACT-C had been culturally adapted and validated in the Serbian language. Given its conciseness, clinical utility, and coverage of physical, emotional, and social domains, we considered it important to expand its applicability by validating it in Serbian.

This study aimed to translate, culturally adapt, and psychometrically validate the CCF-CaQL questionnaire for Serbian-speaking colorectal cancer patients, addressing the need for reliable, culturally relevant QoL assessments for individuals with colorectal cancer in Serbia.

## 2. Materials and Methods

The validation of CCF-CaQL questionnaire was part of the prospective cohort study conducted at the Clinic for Digestive Surgery, located at the University Clinical Center of Serbia, which is the largest university hospital in the country and serves approximately 1.6 million residents of Belgrade and surrounding areas. The study included patients admitted for colorectal carcinoma treatment between 31 May 2022 and 28 February 2023.

A total of 153 patients were approached to participate in the study. Of these, two declined to participate and one could not communicate effectively, resulting in 150 patients included in the final analysis.

Inclusion criteria were adults aged 18 years and older diagnosed with colorectal adenocarcinoma who were scheduled for colorectal surgery. All participants were native Serbian speakers, and those with derived stoma resulting from obstructive colorectal cancer were also included. Patients who provided written informed consent were included in the study.

Exclusion criteria encompassed individuals under 18 years of age, those with inflammatory bowel disease; patients undergoing colorectal surgeries for conditions like diverticulitis and benign polyps, ischemic colitis, hereditary colorectal neoplasia, and recurrent adenocarcinoma; those presenting with metastases; patients unable or unwilling to provide informed consent; and patients unable to communicate effectively due to cognitive impairments. Cognitive impairment was assessed based on clinical judgment made by the attending physician at the time of admission. Patients with visible signs of confusion, disorientation, or a known diagnosis of dementia or delirium, as documented in the medical record, were excluded from the study.

All patients were recruited preoperatively during the admission process for elective colorectal cancer surgery. The questionnaires were administered before surgery. Patients completed the questionnaires independently. However, if any item was unclear, patients were allowed to ask the surveyor for clarification, without leading or influencing their responses. Patients completed the questionnaire at three time points: preoperatively (baseline), one month after surgery, and three months after surgery.

### 2.1. Questionnaires

#### 2.1.1. CCF-CaQL

CCF-CaQL: The CCF-CaQL questionnaire was evaluated in a comprehensive study designed to validate its effectiveness in assessing quality of life among colorectal cancer patients. The study was conducted across multiple international sites, including the United States, France, Germany, and Australia, and involved patients undergoing sphincter-saving surgery for low rectal cancer. It comprises 24 questions. Initially, each question within the questionnaire is assigned a specific point value based on the respondent’s answer. These points are then summed to calculate scores for distinct content measures within the questionnaire. The physical health measure includes questions 6d, 7, 8, 14d, 14e, 15, 16, 19d, 19e, and 20, with a sum score ranging from 0 to 42. The physical activity measure covers questions 6a, 6b, 6c, 17, and 24, with a sum score ranging from 0 to 46. The emotional measure encompasses questions 14a, 14b, 14c, 18, 19a, 19b, 19c, 19f, 21, 22, and 23, with a sum score ranging from 0 to 44. The social measure includes questions 9, 10, 11, 12, and 13, with a total score ranging from 0 to 20. The physical health measure and physical activity measure scores are combined to form the physical element score, which can range from 0 to 88. Similarly, scores of the emotional measure and social measure are summed to calculate the mental element score, which has a potential range of 0 to 64. Ultimately, the physical element and mental element scores are added to determine the total score of the questionnaire, which spans from 0 to 152 [12].

#### 2.1.2. FACT-C

FACT-C: This questionnaire is specifically tailored for cancer patients. It utilizes a five-point Likert scale for each item, ranging from “Not at all” (0) to “Very much”, to capture patients’ quality of life over the past seven days. The questionnaire is organized into five domains: Physical Well-Being (PWB) with 7 items, Social/Family Well-Being (SWB) with 7 items, Emotional Well-Being (EWB) with 6 items, Functional Well-Being (FWB) with 7 items, and the Colorectal Cancer Subscale (CCS) with 9 items. However, the final two items of the CCS, which pertain to ostomy appliances, are not scored as they are relevant only to a minority of patients with a colostomy. Scores for the PWB and EWB domains are calculated in reverse. Domain scores are derived by summing all item scores within each domain, multiplying by the number of items, and dividing by the number of items responded to. To compute the FACT-C total score, domain scores are summed, with possible values ranging from 0 (indicating the worst possible quality of life) to 136 (indicating the best possible quality of life). The FACT-C questionnaire was selected for concurrent validity testing as it is a widely used and validated instrument specifically designed for patients with colorectal cancer. At the time of the study, it was the only colorectal cancer-specific quality of life questionnaire available in a validated Serbian version [11,14].

#### 2.1.3. Sociodemographic Questionnaire

A structured questionnaire was used to collect sociodemographic and clinical data, including age, sex, education level, marital status, employment status, smoking, alcohol consumption, weight, height, and clinical data such as tumor localization, comorbidity, ASA score, stoma status, and neoadjuvant treatment history. The Charlson comorbidity index was used to assess comorbidity burden, and the ASA physical status classification was applied to preoperatively assess patient fitness for surgery [15,16]. The ASA score is determined by skilled anesthesiologist during preoperative check-up.

### 2.2. Translation and Cultural Adaptation of the CCF-CaQL

The translation process was carried out according to the EORTC Quality of Life Group’s recommended steps [17]. The validation process of the CCF-CaQL questionnaire in Serbian involved a meticulous translation protocol to ensure accuracy and cultural relevance. Initially, two forward translations were completed by native Serbian speakers who were also proficient in English. These translations were then reconciled by a third party, who merged the best elements of both versions to enhance clarity and appropriateness. Following this, two proficient English speakers conducted back-translations into English to check the reliability of the translation. An external proofreader subsequently reviewed the preliminary translation. Any feedback received was extensively discussed until a consensus was reached on all aspects. Colorectal surgeons, epidemiologists, and language professionals participated in the translation of the questionnaire. Discrepancies were discussed and resolved through a structured consensus process, ensuring both clinical relevance and cultural appropriateness of the Serbian version. For linguistic validation, the translation was pilot-tested with 15 patients to assess its comprehensibility. Patients reported no difficulties or comments, indicating a successful adaptation. A supplementary table (Supplementary Materials) provides a side-by-side comparison of the original English and translated Serbian items of the CCF-CaQL questionnaire.

### 2.3. Statistical Analysis

Data are presented as mean ± SD for continuous variables and number (percentage) for categorical variables. For the comparison of CCF-CaQL measures, elements, and total between-groups score, an independent T test was used. The *p*-values less than 0.05 were considered statistically significant. At scale level, floor and ceiling effects were considered to be present if more than 15% of respondents achieved, respectively, the lowest or the highest possible score [18]. Repeated measures analysis of variance (ANOVA) was conducted to examine changes in quality of life scores over time for each domain and the total score. Mauchly’s test of sphericity was used to assess the assumption of sphericity. Where this assumption was violated, Greenhouse–Geisser correction was applied. Bonferroni-adjusted pairwise comparisons were performed to identify specific differences between time points. The statistical analyses were performed using SPSS version 26.0 software (SPSS Inc., Chicago, IL, USA) and R 4.3.2. (R Core Team (2023). R is a language and environment for statistical computing from the R Foundation for Statistical Computing, Vienna, Austria (available online: https://www.R-project.org/). The sample size of 150 patients was determined based on standard recommendations for psychometric studies, aiming for at 4–10 participants per item of the questionnaire [19,20]. This provides adequate power for assessing reliability and validity parameters such as internal consistency and construct validity.

#### 2.3.1. Construct Validity

For construct validity, Cronbach’s alpha coefficients were calculated to assess internal consistency. Also, Cronbach’s alpha coefficients if a specific item was deleted were calculated for all measures, elements, and for the total score. A value above 0.70 was considered acceptable [21].

#### 2.3.2. Reliability

The reliability of the CCF-CaQL questionnaire was assessed using split-half reliability analysis [22]. This technique measures the consistency of responses by dividing the test into two halves. For this analysis, items were divided into odd and even groups. Total scores for each half were calculated for every participant, and the Pearson correlation coefficient was used to assess the initial correlation between these scores. This correlation was then used to compute the split-half reliability, using the Spearman–Brown prophecy formula [23].

#### 2.3.3. Concurrent Validity

The concurrent validity of the CCF-CaQL questionnaire was assessed by comparing it to the FACT-C questionnaire. This evaluation involved calculating Pearson’s correlation coefficients to measure the relationships between corresponding scales of the CCF-CaQL and FACT-C [24].

#### 2.3.4. Known-Groups Validity

Clinical validity of the instrument was evaluated using the known-group method [25]. This approach involved comparing different patient groups categorized based on tumor location (colon and rectum), the presence of a stoma, and whether they had received neoadjuvant therapy.

The study was conducted in accordance with the Declaration of Helsinki and was approved by the Ethics Committee of The University Clinical Center Of Serbia (protocol code 808/5, 30 May 2022.).

Informed consent was obtained from all subjects involved in the study.

## 3. Results

The study sample included 150 patients. The sociodemographic and clinical characteristics of included patients are presented in Table 1. The mean age was 64.7 years, with a slight male majority (53.3%). The mean Charlson comorbidity index was 4.4, with 44.7% having moderate and 46.7% severe comorbidity. ASA scores indicated that most patients were either II (54.7%) or III (38.0%). Out of the total, 50.6% of patients had a tumor located in the colon, 48.7% in the rectum, and one patient had two tumors located both in colon and rectum (0.7%). Neoadjuvant treatments were administered to 24.7% for radiotherapy and 26% for chemotherapy, with only 10.7% requiring a stoma. All 150 questionnaires included in the analysis were fully completed. There were no partially completed or incorrectly filled-out questionnaires, and no cases of missing data.

The CCF-CaQL’s structure and internal consistency across various components are presented in Table 2. The physical health measure and physical activity measure had mean scores of 30.6 and 20.2, with no floor or ceiling effects and Cronbach’s alphas of 0.66 and 0.42, respectively. The emotional measure showed a higher internal consistency (α = 0.80) with a mean score of 32.5, alongside a minor ceiling effect of 2.7%. The social measure reported a mean of 12.1 with floor and ceiling effects of 1.3% and 5.3%, respectively, and high internal consistency (α = 0.80). Combining these components, the physical element had a mean score of 50.8 (α = 0.66) and a mental element of 44.6 (α = 0.84), both showing no floor or ceiling effects. The total score was a mean of 95.4 with excellent internal consistency (α = 0.85) and no floor or ceiling effects.

In addition to evaluating the CCF-CaQL, internal consistency was assessed for the FACT-C questionnaire. Cronbach’s alpha values were as follows: PWB = 0.803; SWB = 0.869; EWB = 0.621; FWB = 0.863; CCS = 0.269; FACT-C TOI = 0.491; FACT-G total score = 0.710; and FACT-C total score = 0.691.

The total score demonstrated the highest split-half reliability with a coefficient of 0.94, followed by the mental element and emotional measure, which exhibited split-half reliabilities of 0.899 and 0.893, respectively. The social measure and physical element also showed good split-half reliability with coefficients of 0.86 and 0.87, respectively, while the physical health measure was similarly reliable at 0.86. In contrast, the physical activity measure reported a considerably lower split-half reliability coefficient of 0.512.

The analysis of the impact on Cronbach’s alpha values when individual items are deleted from each scale of the CCF-CaQL questionnaire reveals varying effects across the scales (Table 3). For the physical health measure, deleting certain items results in alpha values ranging from 0.61 to 0.73. The physical activity measure demonstrates a wider range of alpha values from 0.24 to 0.54 upon item deletion. In contrast, the emotional measure and social measure exhibit strong stability, with alpha values consistently between 0.76 and 0.81. The composite scales for the physical element and mental element also show minimal variation in alpha values (0.62 to 0.65 and 0.82 to 0.86, respectively). The overall total score maintains high internal consistency with alpha values steady between 0.83 and 0.87.

The concurrent validity of the CCF-CaQL questionnaire against various FACT-C scales demonstrates significant correlations across multiple dimensions, affirming the robust validity of the CCF-CaQL, particularly for physical health aspects (Table 4). The physical health measure and physical activity measure show strong correlations with FACT-C’s PWB (r = 0.378 and r = 0.430, respectively), EWB (r = 0.308 and r = 0.355, respectively), FWB (r = 0.283 and r = 0.534, respectively), and total score dimensions (r = 0.359 and r = 0.498, respectively). Emotional and social measures also display notable correlations, particularly with the EWB scores, at r = 0.679 and r = 0.574, respectively, underscoring their importance in evaluating emotional well-being. The mental element exhibits the highest correlation with EWB (r = 0.745). The total score of the CCF-CaQL consistently shows strong correlations across all FACT dimensions, especially with FWB (r = 0.566), FACT-G total score (r = 0.658), and FACT-C TOI (r = 0.631).

Table 5 evaluates the known-groups validity of the CCF-CaQL questionnaire by comparing scores across different patient groups based on tumor location, stoma status, and neoadjuvant therapy. Specifically, the physical health measure shows significant differences among patients with tumors in the colon versus the rectum (31.8 ± 5.2 vs. 29.5 ± 6.1, *p* = 0.012) and between those with and without a stoma (31.0 ± 5.4 vs. 26.8 ± 7.9, *p* = 0.005), as well as neoadjuvant therapy recipients compared to those who did not receive this therapy (31.5 ± 5.4 vs. 27.9 ± 6.2, *p* = 0.001). The physical element also indicates that patients with tumors located in the rectum, those with a stoma, and those who received neoadjuvant therapy exhibited poorer physical health outcomes. Additionally, the total score indicated statistically significant variations across tumor location (98.7 ± 16.6 vs. 92.4 ± 19.0, *p* = 0.031), stoma status (96.7 ± 17.9 vs. 85.1 ± 16.5, *p* = 0.015), and neoadjuvant therapy (97.2 ± 17.7 vs. 90.4 ± 18.3, *p* = 0.041).

Longitudinal analysis showed significant changes in CCF-CaQL scores across the three time points: baseline (preoperative), one month, and three months after surgery (Table 6). Physical health declined significantly at one month post operation (30.6 ± 5.8 to 27.6 ± 5.6), followed by partial recovery at three months (29.1 ± 4.4) (overall ANOVA *p* < 0.001; all pairwise comparisons *p* < 0.001). Physical activity, in contrast, showed a continuous increase over time (*p* < 0.001). Emotional and social scores remained stable at one month but improved significantly by three months. The physical and mental composite elements, along with the total CCF-CaQL score, also improved significantly by three months (*p* < 0.001).

## 4. Discussion

Given the substantial and unique impact of colorectal cancer on quality of life, there is a growing need for specific instruments to assess these detailed changes in patients with colorectal cancer. Our objective was to translate, culturally adapt, and psychometrically validate the newly developed CCF-CaQL questionnaire.

The instrument was well accepted by patients, with a high response rate and no missing data, indicating its clarity and ease of use. The demographic and clinical profile of our cohort reveals a typical distribution of colorectal cancer characteristics, which is reflective of wider epidemiological trends [1].

The CCF-CaQL demonstrated strong internal consistency at the total score level, comparable to findings from its original and Turkish versions [12,13]. Emotional and social subscales showed high reliability, while the physical activity subscale yielded lower internal consistency, which is consistent with previous research. This suggests the need for the potential refinement of that domain to improve its psychometric robustness across different populations. Minimal floor and ceiling effects were observed, supporting the questionnaire’s ability to capture diverse patient experiences without response clustering [18,20].

The analysis of Cronbach’s alpha when individual items were deleted showed varied effects across CCF-CaQL scales. In the physical health domain, alpha values ranged from 0.61 to 0.73, with item 6d (medication use for bowel regulation) reducing consistency, suggesting it may not align well with the construct. The physical activity scale showed lower reliability (α = 0.24–0.54), indicating potential for refinement. In contrast, the emotional and social measures were stable and reliable (α = 0.76–0.81), as were the broader physical and mental elements. Overall, the total score remained highly consistent (α = 0.83–0.87), supporting the questionnaire’s reliability.

Concurrent validity was confirmed through moderate to strong correlations with corresponding FACT-C domains. Notably, the CCF-CaQL’s emotional and physical components aligned well with FACT-C’s emotional well-being and functional domains, indicating its effectiveness in capturing core aspects of quality of life in this patient population. Comparing these results with the original study and Turkish validation shows that the CCF-CaQL maintains consistent performance across diverse patient populations [12,13].

Significant differences in physical health measures, physical elements, and the total score between colon and rectum tumor locations (*p* = 0.012, *p* = 0.026, and *p* = 0.031, respectively) align with the existing literature, suggesting location-specific differences in patient outcomes. Studies indicate that the physical and psychological impacts of colorectal cancer may vary significantly depending on the tumor’s location, often due to differences in treatment modalities and tumor behavior [26,27]. The presence of a stoma significantly affected physical health scores and total scores, which is supported by a different study that underscores the profound impact of stoma formation on patients’ quality of life [28]. In contrast to other studies, we did not show the influence of stoma on the mental element [26,29]. Patients receiving neoadjuvant therapy showed notably poorer physical health outcomes and total score compared to those who did not receive such therapy. These findings align with those reported in the original study, where similar impacts were observed [12]. The ability of the CCF-CaQL to distinguish these differences highlights its potential utility in clinical practice, allowing for more tailored interventions and precise monitoring of patient outcomes. This specificity and responsiveness make the CCF-CaQL a valuable tool in both clinical and research settings, aligning with the increasing emphasis on patient-centered care in oncology [30]. Overall, these findings support the clinical applicability of the CCF-CaQL and align with broader research that underscores the importance of using validated patient-reported outcome measures (PROMs) to enhance the quality of care in oncology [30].

As expected, physical health scores declined significantly at one month following surgery, reflecting the immediate impact of surgical recovery, pain, and functional limitations. By three months, most domains—including physical, emotional, and social measures—showed significant improvement, supporting the questionnaire’s sensitivity to changes over time. This trend aligns with the prior literature demonstrating early postoperative declines in QoL, followed by gradual recovery within the first 3 to 6 months post operation [31,32]. The significant changes observed across multiple time points confirm the instrument’s utility for follow-up evaluation in colorectal cancer patients.

Based on its demonstrated reliability and validity, the Serbian version of the CCF-CaQL can serve as a practical tool for healthcare professionals in both clinical and research settings. It can be used to assess baseline quality of life prior to surgery, identify patients in need of additional support (e.g., those with emotional or social distress), monitor patient outcomes across different treatment phases, and guide personalized interventions.

### Strength and Limitations

Key strengths include thorough translation, adaptation, validation, and the inclusion of a diverse patient group. The main limitation was the single-center design. Potential selection bias should be considered, as participants were recruited from a single tertiary surgical center that may not fully reflect the diversity of colorectal cancer patients across Serbia, particularly those treated in regional or lower-resource settings. Patients from remote or underserved areas might be underrepresented. which may limit the broader generalizability of the findings. However, the Clinic for Digestive Surgery at the University Clinical Center of Serbia is a leading institution for colorectal cancer treatment in Serbia. Serving a wide range of patients from various regions and socioeconomic backgrounds across Serbia, the clinic enhances the representativeness of the study, alleviating concerns about the generalizability of the findings to the broader Serbian population. Additionally, the study did not include a test–retest reliability assessment as patients underwent colorectal surgery soon after completing the questionnaires. It was not possible to expect consistent responses due to significant changes in patients’ health status post-surgery. Moreover, while the questionnaire was carefully translated and culturally adapted following international guidelines, subtle cultural nuances in how quality of life is perceived and expressed may influence responses. These cultural dimensions could affect item interpretation and may limit the generalizability of findings beyond the Serbian context. As only patients without metastases scheduled for surgery were included, the findings may not be generalizable to those managed with non-surgical treatments such as palliative care.

## 5. Conclusions

This study successfully translated, culturally adapted, and psychometrically validated the Serbian version of the CCF-CaQL questionnaire in a sample of patients with colorectal cancer. The instrument demonstrated strong internal consistency, acceptable reliability, and good concurrent and known-groups validity. These results suggest that the Serbian version of the CCF-CaQL is a reliable and relevant tool for assessing quality of life in colorectal cancer patients prior to surgery.

## Figures and Tables

**Table 1:** Sociodemographic and clinical characteristics of patients.

	n (%)
Age (mean ± SD)	64.7 ± 10.8
Sex	
Male	80 (53.3)
Female	70 (46.7)
BMI	25.99 ± 4.75
<25	71 (47.3)
25–29.9	46 (30.7)
≥30	33 (22.0)
Education	
Elementary school	14 (9.3)
High school	101 (67.3)
Faculty	35 (23.3)
Marital status	
Married	111 (74.0)
Divorced	12 (8.0)
Unmarried	7 (4.7)
Widowed	20 (13.3)
Employment	
Unemployed	8 (5.3)
Employed	47 (31.3)
Retired	95 (63.3)
Smoking status	
Nonsmoker	64 (42.7)
Previous smoker	54 (36.0)
Current smoker	32 (21.3)
Alcohol consumption	
No	76 (50.7)
Rarely	14 (9.3)
A few times a month	22 (14.7)
At least once a week	24 (16.0)
Daily	14 (9.3)
Charlson comorbidity index (mean ± SD)	4.4 ± 1.3
Mild (CCI scores of 1–2)	13 (8.7)
Moderate (CCI scores of 3–4)	67 (44.7)
Severe (CCI scores ≥5)	70 (46.7)
ASA score	
I	7 (4.7)
II	82 (54.7)
III	57 (38.0)
IV	4 (2.7)
Tumor localization	
Colon	76 (50.6)
Rectum	73 (48.7)
Both	1 (0.7)
Neoadjuvant radiotherapy	37 (24.7)
Neoadjuvant chemotherapy	39 (26.0)
Stoma	
No	134 (89.3)
Yes	16 (10.7)

SD—Standard Deviation; BMI—body mass index, CCI—Charlson comorbidity index; ASA—American Society of Anesthesiologists.

**Table 2:** CCF-CaQL questionnaire structure, floor and ceiling effects, internal consistency, and split-half reliability.

Scaling	*n*	Components	Mean	SD	Range	% Floor	% Ceiling	α	Split-Half Reliability
Physical health measure	150	6d, 7, 8, 14d, 14e, 15, 16, 19d, 19e, 20	30.6	5.8	0–42	0.0	0.0	0.66	0.86
Physical activity measure	150	1, 2, 3, 4, 5, 6a, 6b, 6c, 17, 24	20.2	5.3	0–46	0.0	0.0	0.42	0.51
Emotional measure	150	14a, 14b, 14c, 18, 19a, 19b, 19c, 19f, 21, 22, 23	32.5	7.6	0–44	0.0	2.7	0.80	0.89
Social measure	150	9, 10, 11, 12, 13	12.1	4.8	0–20	1.3	5.3	0.80	0.86
Physical element	150	Physical health measure + Physical activity measure	50.8	9.2	0–88	0.0	0.0	0.66	0.87
Mental element	150	Emotional measure + Social measure	44.6	10.7	0–64	0.0	1.3	0.84	0.90
Total score	150	Physical element + Mental element	95.4	18.1	0–152	0.0	0.0	0.85	0.94

SD—standard deviation; % floor—proportion of patients who scored at the lowest possible score; % ceiling—proportion of patients who scored at the highest possible score; α—Cronbach’s alpha.

**Table 3:** Overview of Cronbach’s alpha values if individual items are deleted from the CCF-CaQL questionnaire scales.

Item	Physical Health Measure α (del) *	Item	Physical Activity Measure α (del) *	Item	Emotional Measure α (del) *	Item	Social Measure α (del) *	Item	Physical Element α (del) *	Item	Mental Element α (del) *	Item	Total Score α (del) *
6d	0.73	1	0.24	14a	0.76	9	0.79	6d	0.68	14a	0.83	6d	0.86
7	0.63	2	0.40	14b	0.76	10	0.76	7	0.64	14b	0.83	7	0.85
8	0.66	3	0.29	14c	0.78	11	0.75	8	0.65	14c	0.83	8	0.85
14d	0.61	4	0.26	18	0.80	12	0.74	14d	0.63	18	0.83	14d	0.85
14e	0.61	5	0.29	19a	0.78	13	0.78	14e	0.63	19a	0.84	14e	0.85
15	0.62	6a	0.49	19b	0.79			15	0.63	19b	0.82	15	0.85
16	0.62	6b	0.53	19c	0.79			16	0.63	19c	0.82	16	0.85
19d	0.61	6c	0.54	19f	0.79			19d	0.64	19f	0.83	19d	0.85
19e	0.61	17	0.33	21	0.80			19e	0.64	21	0.84	19e	0.85
20	0.65	24	0.37	22	0.79			20	0.65	22	0.83	20	0.85
				23	0.81			1	0.62	23	0.83	1	0.84
								2	0.66	9	0.84	2	0.85
								3	0.62	10	0.84	3	0.85
								4	0.62	11	0.84	4	0.85
								5	0.63	12	0.83	5	0.85
								6a	0.70	13	0.84	6a	0.86
								6b	0.70			6b	0.87
								6c	0.71			6c	0.87
								17	0.63			17	0.85
								24	0.65			24	0.85
												14a	0.84
												14b	0.84
												14c	0.85
												18	0.85
												19a	0.85
												19b	0.85
												19c	0.85
												19f	0.85
												21	0.85
												22	0.85
												23	0.85
												9	0.84
												10	0.84
												11	0.84
												12	0.85
												13	0.85

* Cronbach’s alpha if deleted.

**Table 4:** Concurrent validity (Pearson’s correlation) of the CCF-CaQL questionnaire.

CCF-CaQL	FACT-C Questionnaire							
	PWB	SWB	EWB	FWB	CCS	FACT-C Total Score	FACT-G Total Score	FACT-C TOI
Physical health measure	0.378 **	0.085	0.308 **	0.283 **	0.212 **	0.359 **	0.361 **	0.363 **
Physical activity measure	0.430 **	0.151	0.355 **	0.534 **	0.223 **	0.498 **	0.515 **	0.495 **
Emotional measure	0.414 **	0.077	0.679 **	0.453 **	0.172 *	0.435 **	0.548 **	0.510 **
Social measure	0.489 **	0.215 **	0.574 **	0.470 **	0.248 **	0.502 **	0.599 **	0.573 **
Physical element	0.488 **	0.141	0.401 **	0.488 **	0.263 **	0.515 **	0.526 **	0.516 **
Mental element	0.517 **	0.142	0.745 **	0.538 **	0.238 **	0.540 **	0.660 **	0.622 **
Total score	0.554 **	0.156	0.645 **	0.566 **	0.275 **	0.582 **	0.658 **	0.631 **

** Correlation is significant at the 0.01 level; * Correlation is significant at the 0.05 level; PWB, Physical Well-Being; SWB, Social/Family Well-Being; EWB, Emotional Well-Being; FWB, Functional Well-Being; CCS, Colorectal Cancer Subscale; FACT-C, Functional Assessment of Cancer Therapy—Colorectal; FACT-G, Functional Assessment of Cancer Therapy—General; TOI, trial outcome index.

**Table 5:** Known groups validity of CCF questionnaire.

	Tumor Location			Stoma			Neoadjuvant Therapy		
	Colon (*n* = 76)	Rectum (*n* = 73)	*p*	Yes (*n* = 16)	No (*n* = 134)	*p*	Yes (*n* = 39)	No (*n* = 111)	*p*
Physical health measure	31.8 ± 5.2	29.5 ± 6.1	**0.012**	31.0 ± 5.4	26.8 ± 7.9	**0.005**	31.5 ± 5.4	27.9 ± 6.2	**0.001**
Physical activity measure	20.7 ± 5.1	19.7 ± 5.6	0.267	20.5 ± 5.2	17.9 ± 5.5	0.061	20.3 ± 5.3	20.1 ± 5.3	0.811
Emotional measure	33.6 ± 7.3	31.3 ± 7.8	0.062	32.8 ± 7.8	29.6 ± 5.4	0.107	33.0 ± 7.4	30.9 ± 8.0	0.123
Social measure	12.5 ± 4.7	11.8 ± 4.8	0.351	12.3 ± 4.9	10.9 ± 3.8	0.292	12.4 ± 5.0	11.5 ± 4.1	0.349
Physical element	52.5 ± 8.2	49.2 ± 9.8	**0.026**	51.5 ± 8.7	44.6 ± 10.9	**0.004**	51.8 ± 8.7	47.9 ± 10.0	**0.022**
Mental element	46.2 ± 10.2	43.2 ± 11.0	0.086	45.1 ± 10.9	40.5 ± 7.8	0.102	45.4 ± 10.7	42.5 ± 10.4	0.142
Total score	98.7 ± 16.6	92.4 ± 19.0	**0.031**	96.7 ± 17.9	85.1 ± 16.5	**0.015**	97.2 ± 17.7	90.4 ± 18.3	**0.041**

**Table 6:** Changes in CCF-CaQL scores at baseline (before surgery), 1 month, and 3 months after surgery.

	Baseline Testing Before Surgery Mean ± SD	One Month After Surgery Mean ± SD	Three Months After Surgery Mean ± SD	*p* Value
Physical health measure	30.6 ± 5.8	27.6 ± 5.6	29.1 ± 4.4	<0.001 ^a^, <0.001 ^b^, <0.001 ^c^, <0.001 ^d^
Physical activity measure	20.2 ± 5.3	21.3 ± 5.6	26.6 ± 4.1	<0.001 ^a^, 0.003 ^b^, <0.001 ^c^, <0.001 ^d^
Emotional measure	32.5 ± 7.6	32.5 ± 7.3	35.8 ± 6.2	<0.001 ^a^, 1.000 ^b^, <0.001 ^c^, <0.001 ^d^
Social measure	12.1 ± 4.8	12.9 ± 4.7	14.2 ± 4.4	<0.001 ^a^, 0.002 ^b^, <0.001 ^c^, <0.001 ^d^
Physical element	50.8 ± 9.2	48.9 ± 9.2	55.7 ± 6.0	<0.001 ^a^, 0.002 ^b^, <0.001 ^c^, <0.001 ^d^
Mental element	44.6 ± 10.7	45.4 ± 10.5	50.0 ± 9.3	<0.001 ^a^, 0.304 ^b^, <0.001 ^c^, <0.001 ^d^
Total score	95.4 ± 18.1	94.3 ± 17.7	105.7 ± 13.1	<0.001 ^a^, 0.715 ^b^, <0.001 ^c^, <0.001 ^d^

*p*-values represent: a, overall repeated measures ANOVA; b, baseline vs. 1 month; c, baseline vs. 3 months; and d, 1 month vs. 3 months. Pairwise comparisons were adjusted using the Bonferroni method.

## Data Availability

The data presented in this study are available on request from the corresponding author due to privacy reasons.

## Supplementary Materials

The following supporting information can be downloaded at: https://www.mdpi.com/article/10.3390/healthcare13080937/s1. File S1: A side-by-side comparison of the original English and translated Serbian items of the CCF-CaQL questionnaire.

## Abbreviations

The following abbreviations are used in this manuscript:

QoL	Quality of life
CCF-CaQL	Cleveland Clinic Colorectal Cancer Quality of Life Questionnaire
CRC	Colorectal cancer
EORTC	European Organisation for Research and Treatment of Cancer
QLQ-CR29	Quality of Life Questionnaire for Colorectal Cancer
FACIT	Functional Assessment of Cancer Therapy
PWB	Physical Well-Being
SWB	Social/Family Well-Being
EWB	Emotional Well-Being
FWB	Functional Well-Being
CCS	Colorectal Cancer Subscale
SD	Standard deviation
BMI	Body mass index
ASA	American Society of Anesthesiology
PROMs	Patient-reported outcome measures

## References

[1] Bray F., Laversanne M., Sung H., Ferlay J., Siegel R.L., Soerjomataram I., Jemal A. (2024). Global cancer statistics 2022: GLOBOCAN estimates of incidence and mortality worldwide for 36 cancers in 185 countries. CA Cancer J. Clin..

[2] Morgan E., Arnold M., Gini A., Lorenzoni V., Cabasag C.J., Laversanne M., Vignat J., Ferlay J., Murphy N., Bray F. (2023). Global burden of colorectal cancer in 2020 and 2040: Incidence and mortality estimates from GLOBOCAN. Gut.

[3] Siegel R.L., Torre L.A., Soerjomataram I., Hayes R.B., Bray F., Weber T.K., Jemal A. (2019). Global patterns and trends in colorectal cancer incidence in young adults. Gut.

[4] O’Sullivan D.E., Ruan Y., Cheung W.Y., Forbes N., Heitman S.J., Hilsden R.J., Brenner D.R. (2022). Early-onset colorectal cancer incidence, staging, and mortality in Canada: Implications for population-based screening. Am. J. Gastroenterol..

[5] Cancer Serbian Registry (2023). Malignant Tumours in Republic of Serbia. Institute of Public Health of Serbia “Dr Milan Jovanović Batut” Department for Prevention and Control of Noncommunicable Diseases. https://www.batut.org.rs/download/publikacije/MaligniTumoriURepubliciSrbiji2021.pdf.

[6] Todorovic J., Stamenkovic Z., Stevanovic A., Terzic N., Kissimova-Skarbek K., Tozija F., Mechili E.A., Devleesschauwer B., Terzic-Supic Z., Vasic M. (2023). The burden of breast, cervical, and colon and rectum cancer in the Balkan countries, 1990–2019 and forecast to 2030. Arch. Public Health.

[7] Lee L., Dumitra T., Fiore J.F., Mayo N.E., Feldman L.S. (2015). How well are we measuring postoperative “recovery” after abdominal surgery?. Qual. Life Res..

[8] Van der Hout A., Neijenhuijs K.I., Jansen F., van Uden-Kraan C.F., Aaronson N.K., Groenvold M., Holzner B., Terwee C.B., van de Poll-Franse L.V., Cuijpers P. (2019). Measuring health-related quality of life in colorectal cancer patients: Systematic review of measurement properties of the EORTC QLQ-CR29. Support Care Cancer.

[9] Qedair J.T., Al Qurashi A.A., Alamoudi S., Aga S.S., Hakami A.Y. (2022). Assessment of Quality of Life (QoL) of Colorectal Cancer Patients using QLQ-30 and QLQ-CR29 at King Abdulaziz Medical City, Jeddah, Saudi Arabia. Int. J. Surg. Oncol..

[10] Whistance R.N., Conroy T., Chie W., Costantini A., Sezer O., Koller M., Johnson C., Pilkington S., Arraras J., Ben-Josef E. (2009). Clinical and psychometric validation of the EORTC QLQ-CR29 questionnaire module to assess health-related quality of life in patients with colorectal cancer. Eur. J. Cancer.

[11] Ward W.L., Hahn E.A., Mo F., Hernandez L., Tulsky D.S., Cella D. (1999). Reliability and validity of the Functional Assessment of Cancer Therapy-Colorectal (FACT-C) quality of life instrument. Qual. Life Res..

[12] Zutshi M., Aiello A., Fuerst A., Golcher H., Parc Y., Galandiuk S., Hull T.L., Ruppert R. (2020). Reducing Patient Burden and Improving Data Quality With the New Cleveland Clinic Colorectal Cancer Quality of Life Questionnaire. Dis. Colon Rectum.

[13] Ozata I.H., Tufekci T., Karahan S.N., Sucu S., Yigit D., Ozoran E., Ozturk O., Veznikli M., Baygul A., Demirel A.O. (2023). Reliability and validity of the Turkish version of the New Cleveland Clinic Colorectal Cancer Quality of Life Questionnaire. Int. J. Colorectal Dis..

[14] Ilic-Zivojinovic J., Krdzic I., Jovanovic A., Vukasinovic D., Ilic B., Gavrilovic A., Soldatovic I. (2022). Transcultural adaptation and validation of the Serbian version of the colorectal-specific quality of life questionnaire FACT-C. PLoS ONE.

[15] Charlson M.E., Pompei P., Ales K.L., MacKenzie C.R. (1987). A new method of classifying prognostic comorbidity in longitudinal studies: Development and validation. J. Chronic Dis..

[16] Daabiss M. (2011). American Society of Anaesthesiologists physical status classification. Indian J. Anaesth..

[17] EORTC Quality of Life Group (2017). EORTC Quality of Life Group Translation Procedure.

[18] Terwee C.B., Bot S.D.M., de Boer M.R., van der Windt D.A.W.M., Knol D.L., Dekker J., Bouter L.M., de Vet H.C.W. (2007). Quality criteria were proposed for measurement properties of health status questionnaires. J. Clin. Epidemiol..

[19] Memon M.A., Ting H., Cheah J.-H., Thurasamy R., Chuah F. (2020). Sample size for survey research: Review and recommendations. J. Appl. Struct. Equ. Model..

[20] Burton L.J., Mazerolle S.M. (2011). Survey instrument validity part I: Principles of survey instrument development and validation in athletic training education research. Athl. Train. Educ. J..

[21] Cronbach L.J. (1951). Coefficient alpha and the internal structure of tests. Psychometrika.

[22] Parsons S., Kruijt A.-W., Fox E. (2019). Psychological science needs a standard practice of reporting the reliability of cognitive-behavioral measurements. Adv. Methods Pract. Psychol. Sci..

[23] de Vet H.C.W., Mokkink L.B., Mosmuller D.G., Terwee C.B. (2017). Spearman-Brown prophecy formula and Cronbach’s alpha: Different faces of reliability and opportunities for new applications. J. Clin. Epidemiol..

[24] Lin W.L., Yao G., Michalos A.C. (2014). Concurrent validity. Encyclopedia of Quality of Life and Well-Being Research.

[25] Davidson M., Michalos A.C. (2014). Known-groups validity. Encyclopedia of Quality of Life and Well-Being Research.

[26] Ihn M.H., Lee S.M., Son I.T., Park J.T., Oh H.-K., Kim D.-W., Kang S.-B. (2015). Cultural adaptation and validation of the Korean version of the EORTC QLQ-CR29 in patients with colorectal cancer. Support Care Cancer.

[27] Yacir E.A., Hadj O.M., Hafid H., Said B. (2022). Cultural adaptation and validation of the Moroccan version of the EORTC QLQ-CR29 in patients with colorectal cancer. Asian Pac. J. Cancer Prev..

[28] Vonk-Klaassen S.M., de Vocht H.M., den Ouden M.E., Eddes E.H., Schuurmans M.J. (2016). Ostomy-related problems and their impact on quality of life of colorectal cancer ostomates: A systematic review. Qual. Life Res..

[29] Lin J.B., Zhang L., Wu D.W., Xi Z.-H., Wang X.-J., Lin Y.-S., Fujiwara W., Tian J.-R., Wang M., Peng P. (2017). Validation of the Chinese version of the EORTC QLQ-CR29 in patients with colorectal cancer. World J. Gastroenterol..

[30] Doolin J.W., Halpin M., Berry J.L., Hshieh T., Zerillo J.A. (2020). Why focus on patient-reported outcome measures in older colorectal cancer patients?. Eur. J. Surg. Oncol..

[31] Roh M.S., Colangelo L.H., O’Connell M.J., Yothers G., Deutsch M., Allegra C.J., Kahlenberg M.S., Baez-Diaz L., Ursiny C.S., Petrelli N.J. (2009). Pre-operative multimodality therapy improves disease-free survival in patients with carcinoma of the rectum: NSABP R-03. J. Clin. Oncol..

[32] Montazeri A. (2009). Quality of life data as prognostic indicators of survival in cancer patients: An overview of the literature from 1982 to 2008. Health Qual. Life Outcomes.

